# Risk Factors and Outcomes of Extended Length of Stay in Older Adults with Intertrochanteric Fracture Surgery: A Retrospective Cohort Study of 2132 Patients

**DOI:** 10.3390/jcm11247366

**Published:** 2022-12-12

**Authors:** Yubin Long, Tao Wang, Xin Xu, Guangyuan Ran, Heng Zhang, Qi Dong, Qi Zhang, Junfei Guo, Zhiyong Hou

**Affiliations:** 1 Department of Orthopaedics Surgery, Third Hospital of Hebei Medical University, Shijiazhuang 050051, China; 2 Orthopedic Institute of Hebei Province, Shijiazhuang 050051, China; 3 Department of Orthopaedics Surgery, Baoding First Central Hospital, Baoding 071000, China; 4 Key Laboratory of Medical Biotechnology of Hebei Province, Department of Biochemistry and Molecular Biology, College of Basic Medicine, Cardiovascular Medical Science Center, Hebei Medical University, Shijiazhuang 050011, China; 5 Department of Anesthesiology, Children’s Hospital of Hebei Affiliated to Hebei Medical University, Shijiazhuang 050031, China; 6 NHC Key Laboratory of Intelligent Orthopedic Equipment, Shijiazhuang 050051, China

**Keywords:** hip fracture, intertrochanteric, older adults, extended, length of hospital stay, risk factors, mortality, outcomes

## Abstract

We aimed to identify the risk factors associated with an extended length of hospital stay (eLOS) in older hip-fracture patients and to explore the relationships between eLOS and mortality and functional outcomes. In this retrospective analysis of surgically treated intertrochanteric fracture (IF) patients, all variables were obtained and compared between the eLOS group and the normal LOS group. All participants were followed-up for a minimum of two years and the relation between the eLOS and all-cause mortality and functional outcomes were compared. After adjustment for potential confounders, we identified that patients with high modified Elixhauser’s Comorbidity Measure (mECM) had the highest likelihood of eLOS, followed by obesity, admission in winter, living in urban, pulmonary complications, admission in autumn, and time from injury to surgery. In addition, our results showed no significant difference in the mortality and functional outcomes between the two groups during follow-up. By identifying these risk factors in the Chinese geriatric population, it may be possible to risk-stratify IF patients and subsequently streamline inpatient resource utilization. However, the differences between health care systems must be taken into consideration. Future studies are needed to preemptively target the modifiable risk factors to demonstrate benefits in diminishing eLOS.

## 1. Introduction

Increasing aging and the prevalence of hip fractures will lead to more hip fracture patients requiring surgery and management strategies in the perioperative setting for comorbidities and complications [1]. By 2018, the number of older adults aged 60 and older in China was approximately 250 million, representing nearly a fifth of the population. By 2050, that percentage will rise to close to 450 million, or about 30 percent of the population [2,3]. Worldwide, the number of people aged 65 years and over is expected to reach 16.7% by 2050 and the total number of hip fractures is projected to keep increasing and surpass six million by the year 2050 [4]. Nowadays, intertrochanteric fractures (IF), which are low-violence fractures that are mostly related to osteoporosis, account for almost half of all hip fractures and represent a major public health concern in older adults due to multiple concurrent comorbidities and an extended length of hospital stay (eLOS), ultimately leading to a heavy inpatient hospitalization cost [5].

In the United States, over 250,000 patients are hospitalized each year, and over 60% of the 13.8 billion dollars associated with osteoporotic fractures were attributed to hip fracture health care and is projected to cost over 18.2 billion dollars by 2025 [6,7,8]. Although specialized orthogeriatric co-management by a multidisciplinary team has been shown to prevent perioperative complications and adverse outcomes, costs from hip fractures are still disproportionately borne by elderly patients [9]. Previous studies have demonstrated that hip fracture accounts for 14% of all geriatric fractures, but accounts for about 72% of all health care costs [8,10,11]. The primary reasons are considerable expenditure spent on hospitalization, preoperative patient status optimization, and management of the complications.

Recently, multiple studies have focused on the role of eLOS in the costs of inpatient hip fracture care and implemented continuous efforts to reduce the eLOS [12,13,14,15,16]. Thus, a targeted approach that identifies and targets factors associated with eLOS among this population might be particularly effective to optimize health care resource utilization. Despite substantial progress in identifying risk factors of eLOS over the past few decades, there is still an ongoing debate on whether several variables are key drivers of eLOS. For example, on one hand, there is literature supporting the idea that advanced age contributes to eLOS [17,18,19]. However, some studies have different opinions, and they believe that age is not related to eLOS [10,20,21].

In other words, current research is uncertain whether the identified factors can be applied to trauma centers with large volumes of hip fracture patients. Especially in China, it seems very meaningful since relevant research exploring eLOS risk factors after hip fractures in Chinese is still relatively scarce. The first aim of the present paper was thus to identify the risk factors associated with eLOS in older patients following IF and treated by intramedullary fixation (IMN) in a tertiary hospital in China. The second aim was to explore the relationships between eLOS and patient mortality and functional outcomes.

## 2. Materials and Methods

### 2.1. Study Design, Setting, and Population

A retrospective analysis was conducted on all IF patients who had undergone IMN by proximal femoral nail anti-rotation (PFNA) at an urban, Level I regional trauma center and ranked among the national top 10 between January 2017 and March 2020. Patients with age ≥ 65 years old, diagnosed with IF, and treated with PFNA, having an admission delay within 48 h after the initial injury, and who had received follow-up for at least two years were enrolled in the study. Patients with pathological fractures and fractures of unknown etiology, old fracture (>3 weeks after injury), open fracture, patients with multiple injuries, previous hip fracture surgery, with a LOS longer than 30 days, and patients who were treated conservatively or denied surgery were excluded from the study. Patients were assigned into two groups: the normal LOS group (nLOS) or the eLOS group according to whether they had a hospital stay ≥14 days (both the mean and median values in this study were approximately equal to 14). The study was overseen and approved by the institutional internal review board of the participating institution in compliance with the Declaration of Helsinki and consent was waived as this was an observational study without intervention. All collected patient data were anonymously recorded to protect patient confidentiality.

### 2.2. Perioperative Treatment and Surgical Procedure

Through the application of an integrated management bundle that incorporates multidisciplinary measures, we developed a geriatric orthopedics ward in our hospital, which has been described in detail in our prior literature [22]. At least two orthopedists, an attending anesthesiologist, one general internist, and nurses assess patients every day in wards as part of a multidisciplinary team responsible for their management.

Before surgery, X-rays of the injured leg were taken and additional CT scans carried out depending on the condition of the patient. Following the Orthopedic Trauma Association Classification System, fractures were defined as stable or unstable. All fractures were treated by PFNA under general or regional anesthesia according to the international treatment guidelines. After the surgery, gradually progressive full weight bearing was allowed and encouraged early following internal fixation. Patients or family members were regularly contacted by outpatient review or telephone interview follow-up including rehabilitation guidance.

All patients were allowed to discharge from the hospital when they met the discharge criteria. Discharge criteria included adequate pain control utilizing an oral-analgesics that did not affect the patient’s sleep and functional exercises, tolerating oral intake, and having the ability to self-care. Other discharge criteria included the patients’ vital signs being stable, spirit and appetite had returned to the preoperative level, and stool was normal, the incision was dry with no signs of infection. Physical therapy was initiated within 24–48 h following surgery, and if patients could walk independently or with the help of the walking aid when meeting the discharge criteria, the patients were discharged home.

### 2.3. Data Collection

Using our institution’s electronic medical records, we retrospectively collected demographic information on our patients including gender, age, age group (categorized into 10-year age intervals), body mass index (BMI), place of residence (rural or urban), smoking and drinking status; surgery-related indicators including fracture type, surgical delay (time from initial injury to surgery), the modified Elixhauser comorbidity method (mECM), duration of operation, type of anesthesia, and intraoperative blood loss; and perioperative clinical indicators including numerical rating scores (NRS) at admission [23,24], Geriatric Depression Scale (GDS), functional independence measure (FIM), presence or absence of anxiety, Hb at admission, received a blood transfusion or not, the season of admission, and complications during hospitalization. The survival status and time of each patient were recorded from the date of hospital discharge to mortality or the end of follow-up. Then, mortalities at various times after surgery were also recorded.

### 2.4. Definitions

The ages of the patients were categorized into 10-year groups, while the BMI was grouped as normal (BMI < 24 kg/m^2^), overweight (24 ≤ BMI < 28 kg/m^2^), and obesity (BMI ≥ 28 kg/m^2^). Fracture type was identified according to the AO/OTA classification into stable or unstable. Using the mECM, comorbidities were scored at admission and then stratified into five groups in this research. To assess the depression symptoms and ADL-related generic ability, we used the 15-item GDS and FIM, respectively [25,26]. Breakpoints of 8, 10, and 12 g/dL were used to classify the Hb levels at admission. We regarded March, April, and May as spring; June, July, and August as summer; September, October, and November as autumn; and January, February, and December as winter, according to the average temperature in our city all year round. Perioperative complications classified as severe complications, cardiac complications, pulmonary complications, neurological complications, hematological complications, and nutritional/metabolic complications were also recorded, which were described in detail in our previous paper [27].

### 2.5. Statistical Analysis

We performed a power analysis for statistics. A two-sided 5% significance level and 80% power were considered reliable and significant. The sample size and the power analysis were computed using NCSS-PASS V11.0.7 software valid until 15 October 2022 (https://www.ncss.com/software/pass/). Testing for normality was performed by applying the Shapiro–Wilk test to continuous variables. The enumeration data were expressed as a percentage (%) processed by the chi-square test. The measurement data are expressed as the mean (SD), and Students’ t-tests were used to compare normally distributed samples. Since missing data were assumed to be randomly missing, and the proportion of missing data (≤5%) was small, no data were imputed. An analysis of the univariate and multivariate data was performed encompassing all characteristics found to be independently correlated with eLOS as the candidate predictor variables. Initially, all covariates were accounted for in the univariate analysis, and then all covariates were accounted for with a *p*-value < 0.1 in the multivariable analysis. Furthermore, we looked at the relationship between eLOS and all-cause mortality at various times after surgery. Finally, we constructed the validity for eLOS by taking into account mECM and measuring the association between eLOS with the identified risk factors influenced by mECM. An analysis of the partial correlations between the risk factors and eLOS was conducted. All data analyses were performed using IBM SPSS Statistics for Windows, version 26.0 (IBM, Armonk, NY, USA). A two-sided *p*-value < 0.05 was considered significant.

## 3. Results

**Figure 1:**
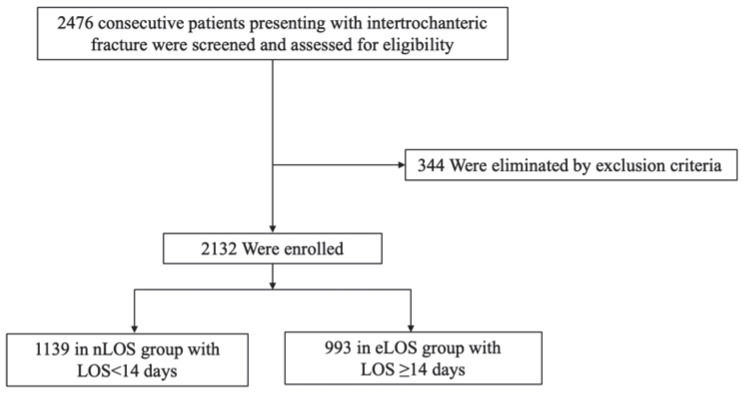
Flow diagram of the included patients.

From January 2017 and March 2020, retrospective reviews of 2476 consecutive IF patients were conducted and ineligible patients were excluded through exclusion criteria in 344 cases. To eliminate the issue of a few individuals with atypical medical conditions impacting the data unduly, we excluded 60 patients having a LOS longer than 30 days; other reasons for exclusion were under 65 years old (66 patients), treated conservatively (123 patients), delayed more than 48 h (114 patients), open or old hip fractures, pathological fractures, previous hip fracture surgery, or multiple injuries (48 patients), and lost to follow-up (203 patients). Finally, a total of 2132 patients were ultimately enrolled in this study, of which 1139 were in the nLOS group and 993 were in the eLOS group (Figure 1). The majority of patients were female (67.7%) and the mean age was 79.0 years (SD 7.2). The average follow-up was 34.8 months. The mean LOS of all participants was 13.9 ± 5.0 days (range 4–30) and the median LOS was 14 days.

The comparisons of the patient characteristics and hospital outcomes in the two groups were presented in Table 1. Univariate analyses showed statistically significant differences between the eLOS group and the nLOS groups (all *p* < 0.10) in 11 variables, which were entered as covariates in the multivariate analysis model. When combined with clinical experience, gender, age, and age group were forced into the model, although not significant. The model thereafter inferred that obesity (with BMI ≥ 28), living in urban areas, time from injury to surgery, mECM, admission in autumn and winter, and pulmonary complications were found to be significantly associated with eLOS. In terms of other dummy variables of BMI and admitted season, the effect sizes of overweight and admitted in spring were small and nonsignificant. On the whole, patients with high mECM grades had the highest likelihood of eLOS. Other predictors with effect sizes from large to small, in order, were obesity, admission in winter, living in urban, pulmonary complications, admission in autumn, and time from injury to surgery. No differences were found in gender, age, age group, intraoperative blood loss, NRS, whether receiving a blood transfusion or not, and other perioperative complications between the two groups (Table 1).

The results of the mortality rates at various times after surgery, functional outcomes, and destination after discharge of the two groups are detailed in Table 2. All patients achieved a minimum 24-month and a maximum 62-month (mean 34.8 months) follow-up. During the follow-up, the overall patient mortality was 22.9% (487 cases). At the end of this study, the overall study case fatality for patients in nLOS and eLOS were 21.7% (248 cases) and 24.2% (239 cases), respectively. Our results showed no significant difference in mortality rates at 1 month, 1–3 months, 3–6 months, 6–12 months, and 12–24 months after surgery and the functional outcomes between the two groups during follow-up (all *p* > 0.05). Almost seventy percent of the patients could be discharged to their homes. An additional group of 19.9% was discharged home after treatment in a rehabilitation clinic. The patients were transferred to other hospitals, rehabilitation facilities, and nursing homes, mainly closer to home, at 7.0%, 11.5%, and 8.9%, respectively.

To test the robustness of these results while such interactions between mECM and other variables were realized, simple correlation and partial correlation analysis were performed, controlling for the mECM. The results revealed statistically significant correlations between the eLOS and variables including residence, time from injury to surgery, the season of admission, and pulmonary complications, however, the strength of most correlations was in the weak range (Table 3).

## 4. Discussion

According to the literature, the average LOS varies widely among different countries from 6.9 to 36.8 days [4,28,29], which may be attributed to differences in health care systems across countries. Chinese, Japanese, Korean, and other Asian populations share a similar health care policy and social environments, assisting with postoperative care and rehabilitation in hospitals and transferring patients to home or non-medical facilities after surgery. Based on our results, the mean or median LOS of this cohort was approximately 14 days. Consequently, it was used as the cutoff point for the definition of eLOS in this study.

This retrospective study involved 2132 participants and was conducted at an urban, Level I regional trauma center and ranked among the national top 10, we identified several perioperative variables associated with eLOS. Patients with high mECM grades had the highest likelihood of eLOS, followed by obesity, admission in winter, living in urban, pulmonary complications, admission in autumn, and time from injury to surgery. Our study supported the conclusions of the previous literature that eLOS is significantly associated with complications [9,30], the American Society of Anesthesiologists (ASA) physical status classification [10], surgical delay [9,20,28,31,32], and pneumonia [30,33,34].

While most previous studies [9,10,30,35,36] have found longer LOS in the hip fracture population and were more likely to be male, we did not find this to have a significant association with eLOS. However, in addition to the previously known risk factors, we also found additional candidates for the eLOS risk factors. Surprisingly, we observed that patients who were admitted in autumn and winter had significantly longer LOS in comparison with the admission season of summer. This finding was consistent with that reported in other comorbidities and complications such as hypertension, cardiovascular disease, renal disease, and respiratory disease [37,38,39,40,41], which were significantly associated with seasonal variation. In particular, large temperature differences throughout all four seasons in our city pose an additional risk of these seasonal variation-related complications because the research region is located in the North China Plain with the maximum air temperatures being up to 42.7 °C during summer, while the minimum air temperatures are −26.5 °C during winter. This is the first study to highlight the effect of admission season as a significant predictor of eLOS following hip fracture. We then speculated that the eLOS was a result of the admission of season-related complications. Moreover, the biologically plausible temperature hypothesis has proven that the cold temperature could elevate systemic vascular resistance and fibrinogen levels, resulting in an increase in blood pressure, thrombus formation as well as enhanced fibrinolytic activity, platelet adhesiveness, and lipid levels [42,43], which may make it easier to cause perioperative complications. Although several of the above-mentioned seasonal-related diseases including hematological complications and cardiac complications were not significantly correlated with eLOS after multivariate adjustment, the trends were observed with significant differences in our univariate analysis. Thus, we also believe that some postoperative cardiovascular and cerebrovascular diseases may be the reason for the close relationship between the season of admission and eLOS.

In addition, Ogawa et al. [44] evaluated the relationship between mortality and season of admission in patients following hip surgery and discovered significantly higher mortality during the autumn and winter. However, there were no significant differences in the mortality rates at 1 month, 1–3 months, 3–6 months, 6–12 months, and 12–24 months after surgery and functional outcomes between the two groups during follow-up. This is due, in part, to the particular management of elderly hip fracture patients who receive standard combination therapy in specialist geriatric trauma orthopedic wards in our hospital, providing 24/7 geriatric support throughout the year. This also leads to the fact that the mean LOS in our cohort was shorter than those reported in recent articles [9,30,45].

Despite other easily identified factors such as obesity, place of residence, the season of admission, and patient physical status in this study not being modifiable, by identifying these non-modifiable factors earlier, we can better project costs and manage expectations. Alternatively or additionally, the most obvious conclusion that deserves full attention in this study was that pulmonary complication, as a modifiable risk factor, has been shown to have the potential to be targeted for preemptive prevention with multiple effective interventions introduced [33,46]. In our study, both univariate and multivariate analyses demonstrated that prolonged time from injury to surgery was identified as an important risk factor for eLOS in this study, which was in agreement with the findings of other researchers [9,32,47]. In a comorbidity-adjusted analysis study by Alvi et al. [47], the researcher stated that surgical delay did not affect the overall complications, readmissions, or mortality after 30 days, but it did increase the LOS. More recently, Hecht et al. [32] found that surgical delays of one day could extend the average LOS by 0.75 days. Therefore, an investigation of possible interventions to reduce unnecessary surgery delays conducted by a multidisciplinary team is the top priority for accelerating surgery when considering preoperative optimization is an integral component of treating these frail patients.

Previously, several researchers have assessed the factors influencing the eLOS and its related adverse outcomes and mortalities in hip fracture patients. The key strengths of the present study are the large sample size of participating units by using more recent data and the consistency and high quality with which the data were collected, compared to the earlier studies. In addition, we involved a specific cohort of patients with intertrochanteric hip fractures who received surgery by a single internal fixation, which eliminated the effects of possible confounding variables. To the best of our knowledge, this is the largest study to investigate factors associated with eLOS in a Chinese population and the first study to identify seasonal effects as a prognostic factor for eLOS in elderly patients with IF. Furthermore, the scores from the sets of scoring systems involved and all of the surgeries used the same protocol and implants, which decreased the effects of some confounding factors. The limitation was the retrospective single-center study design. In addition, other unknown factors including perioperative laboratory values and surgeon volume may also have contributed, which were not assessed or included in the analysis. Finally, the differences between health care systems must be taken into consideration when interpreting our results. However, the study provides valuable information regarding the factors that influence eLOS in older adults with hip fractures.

## 5. Conclusions

By identifying these risk factors, we found that older hip-fractured adults with high mECM grades had the highest likelihood of eLOS, followed by obesity, admission in winter, living in urban area, pulmonary complications, admission in autumn, and time from injury to surgery in the Chinese population. Therefore, it may be possible to risk stratify hip fracture patients and subsequently streamline inpatient resource utilization. Future studies are needed to preemptively target modifiable risk factors to demonstrate the benefits in diminishing eLOS and the total hospital costs.

## Figures and Tables

**Table 1:** The univariate and multivariate analyses of all of the involved possible factors associated with eLOS.

Variables	Univariate				Multivariate	
	Total (*n* = 2132)	nLOS (*n* = 1139)	eLOS (*n* = 993)	*p*-Value ^a^	OR (95%CI)	*p*-Value ^b^
Demographics						
Gender, *n* (%)				0.893		
Male	688 (32.3%)	369 (32.4%)	319 (32.1%)		Reference	
Female	1444 (67.7%)	770 (67.6%)	674 (67.9%)		0.939 (0.766, 1.150)	0.541
Age, years	79.0 ± 7.2	79.1 ± 7.3	79.0 ±7.1	0.724	1.016 (0.980, 1.053)	0.391
Age group, *n* (%)				0.471		
65–69	234 (11.0%)	122 (10.7%)	112 (11.3%)		Reference	
70–79	851 (39.9%)	450 (39.5%)	401 (40.4%)		0.913 (0.598, 1.394)	0.674
80–89	894 (41.9%)	485 (42.6%)	409 (41.2%)		0.714 (0.364, 1.402)	0.328
90–99	146 (6.8%)	76 (6.7%)	70 (7.0%)		0.681 (0.252, 1.842)	0.449
≥100	7 (0.4%)	6 (0.5%)	1 (0.1%)		0.118 (0.010, 1.429)	0.093
BMI, kg/m^2^, *n* (%)				0.076 *		
Normal (BMI < 24)	1390 (65.2%)	757 (66.5%)	633 (63.7%)		Reference	
Overweight (24 ≤ BMI < 28)	580 (27.2%)	309 (27.1%)	271 (27.3%)		1.037 (0.836, 1.285)	0.742
Obesity (BMI ≥ 28)	162 (7.6%)	73 (6.4%)	89 (9.0%)		1.654 (1.153, 2.373)	0.006 *
Residence, *n* (%)				0.001 **		
Rural	749 (35.1%)	437 (38.4%)	312 (31.4%)		Reference	
Urban	1383 (64.9%)	702 (61.6%)	681 (68.6%)		1.512 (1.243, 1,840)	<0.001 *
Smoking status, *n* (%)				0.939		
Never	1757 (82.4%)	941 (82.6%)	816 (82.1%)			
Past	157 (7.4%)	84 (7.4%)	73 (7.4%)			
Current	218 (10.2%)	114 (10.0%)	104 (10.5%)			
Drinking status, *n* (%)				0.990		
Current	86 (4.0%)	46 (4.0%)	40 (4.0%)			
Never	2046 (96.0%)	1093 (96.0%)	953 (96.0%)			
Surgery-related indicators						
Fracture type, *n* (%)				0.143		
Stable (A1.1–A2.1)	1159 (54.4%)	636 (55.8%)	523 (52.7%)			
Unstable (A2.2–A3.3)	973 (45.6%)	503 (44.2%)	470 (47.3%)			
Time from injury to surgery, days	6.0 ± 3.1	4.9 ± 2.1	7.2 ± 3.6	<0.001 **	1.342 (1.293, 1.394)	<0.001 *
mECM, *n* (%)				0.010 **		
<0	44 (2.1%)	30 (2.6%)	14 (1.4%)		Reference	
0	1077 (50.5%)	596 (52.3%)	481 (48.4%)		2.299 (1,145, 4.615)	0.019 *
1–5	349 (16.4%)	182 (16.0%)	167 (16.8%)		1.935 (1.332, 3.973)	0.022 *
6–13	580 (27.2%)	299 (26.3%)	281 (28.3%)		2.071 (1.021, 4.199)	0.044 *
≥14	82 (3.8%)	32 (2.8%)	50 (5.1%)		2.958 (1.277, 6.854)	0.011 *
Type of anesthesia, *n* (%)				0.249		
General	799 (37.5%)	414 (36.3%)	385 (38.8%)			
Regional	1333 (62.5%)	725 (63.7%)	608 (61.2%)			
Duration of operation, min	99.3 ± 34.9	98.3 ± 34.2	100.4 ± 35.7	0.158		
Intraoperative blood loss, mL	238.5 ± 158.1	229.0 ± 159.6	249.4 ± 155.7	0.003 **	1.000 (1.000, 1.001)	0.241
Periopertive clinical indicators						
NRS	5.3 ± 1.8	5.4 ± 1.8	5.2 ± 1.8	0.041 **	0.982 (0.931, 1.036)	0.504
GDS	4.1 ± 1.4	4.1 ± 1.4	4.1 ± 1.4	0.995		
FIM	83.7 ± 10.4	83.8 ± 10.5	83.6 ± 10.2	0.646		
Anxiety, *n* (%)				0.181		
No	1737 (81.5%)	916 (80.4%)	821 (82.7%)			
Yes	395 (18.5%)	223 (19.6%)	172 (17.3%)			
Hb level at admission, g/dL				0.718		
Hb ≥ 12	625 (29.3%)	338 (29.7%)	287 (28.9%)			
12 > Hb ≥ 10	885 (41.5%)	475 (41.7%)	410 (41.3%)			
10 > Hb ≥ 8	512 (24.0%)	273 (24.0%)	239 (24.1%)			
Hb < 8	110 (5.2%)	53 (4.6%)	57 (5.7%)			
Blood transfusion, n (%)				0.001 **		
No	515 (24.2%)	308 (27.0%)	207 (20.8%)		Reference	
Yes	1617 (75.8%)	831 (73.0%)	786 (79.2%)		1.203 (0.952, 1.519)	0.121
Season of admission, *n* (%)				0.013 **		
Spring	548 (25.7%)	301 (26.4%)	247 (24.9%)		1.107 (0.847, 1.448)	0.456
Summer	484 (22.7%)	285 (25.0%)	199 (20.0%)		Reference	
Autumn	533 (25.0%)	270 (23.7%)	263 (26.5%)		1.390 (1.063, 1.818)	0.016 *
Winter	567 (26.6%)	283 (24.8%)	284 (28.6%)		1.547 (1.186, 2.019)	0.001 *
Complications during hospitalization, *n* (%)						
Severe complications				0.127		
No	1780 (83.5%)	964 (84.6%)	816 (82.2%)			
Yes	352 (16.5%)	175 (15.4%)	177 (17.8%)			
Cardiac complications				0.070 *		
No	1652 (77.5%)	900 (79.0%)	752 (75.7%)		Reference	
Yes	480 (22.5%)	239 (21.0%)	241 (24.3%)		0.995 (0.788, 1.255)	0.964
Pulmonary complications				0.005 **		
No	1926 (90.3%)	1048 (92.0%)	878 (88.4%)		Reference	
Yes	206 (9.7%)	91 (8.0%)	115 (11.6%)		1.451 (1.057, 1.991)	0.021 *
Neurological complications				0.277		
No	1964 (92.1%)	1056 (92.7%)	908 (91.4%)			
Yes	168 (7.9%)	83 (7.3%)	85 (8.6%)			
Hematological complications				0.021 **		
No	1216 (57.0%)	676 (59.4%)	540 (54.4%)		Reference	
Yes	916 (43.0%)	463 (40.6%)	453 (45.6%)		1.060 (0.877, 1.281)	0.545
Endocrine/metabolic complications				0.712		
No	659 (30.9%)	356 (31.3%)	303 (30.5%)			
Yes	1473 (69.1%)	783 (68.7%)	690 (69.5%)			

Values are presented as the number (%) or the mean ± SD (standard deviation). ^a,^* *p* < 0.10: statistical significance ** *p* < 0.05: statistical significance ^b,^* *p* < 0.05: statistical significance. Notes: OR, odds ratio; eLOS, extended length of hospital stay; nLOS, normal length of hospital stay; BMI, body mass index; mECM, modified Elixhauser’s Comorbidity Measure; NRS, numerical rating scores; GDS, Geriatric Depression Scale; FIM, functional independence measure.

**Table 2:** Comparisons of the mortality rates, functional outcomes, and destination after discharge between the nLOS and eLOS groups.

Variables	Total (*n* = 2132)	nLOS (*n* = 1139)	eLOS (*n* = 993)	*p*-Value
Mortality rates (*n*, %)				
1 month	17 (0.8%)	6 (0.5%)	11 (1.1%)	0.132
1–3 months	22 (1.0%)	11 (1.0%)	11 (1.1%)	0.736
3–6 months	27 (1.3%)	17 (1.5%)	10 (1.0%)	0.327
6–12 months	71 (3.4%)	40 (3.6%)	31 (3.2%)	0.624
12–24 months	118 (5.9%)	65 (6.1%)	53 (5.7%)	0.703
Functional Outcomes (*n*, %)				0.408
Independent walking	768 (36.0%)	427 (37.5%)	341 (34.3%)	
Use of walking aids	660 (31.0%)	343 (30.1%)	317 (31.9%)	
Use of wheelchair	133 (6.2%)	76 (6.7%)	57 (5.7%)	
Bedridden	84 (3.9%)	45 (4.0%)	39 (3.9%)	
Death	487 (22.9%)	248 (21.7%)	239 (24.2%)	
Destination after discharge (*n*, %)				0.027 *
Home	1548 (72.6%)	855 (75.1%)	693 (69.8%)	
Other hospitals	150 (7.0%)	78 (6.8%)	72 (7.3%)	
Rehabilitation facilities	245 (11.5%)	121 (10.6%)	124 (12.5%)	
Nursing homes	189 (8.9%)	85 (7.5%)	104 (10.5%)	

Values are presented as the number (%). Mortality rates are reported at various times after surgery. Notes: eLOS, extended length of hospital stay; nLOS, normal length of hospital stay. * *p* < 0.05: statistical significance.

**Table 3:** The association between eLOS with the identified risk factors influenced by patient comorbidities, which are recorded and measured as mECM.

Variables	eLOS			
	Uncontrol for mECM		Control for mECM	
	Spearman’s r Statistic	*p*-Value	Partial Correlation Coefficient	*p*-Value
BMI	−0.021	0.333	−0.018	0.418
Residence	0.073	0.001 *	0.073	0.001 *
Time from injury to surgery	0.328	<0.001 *	0.358	<0.001 *
Season of admission	0.052	0.017 *	0.051	0.018 *
Pulmonary complications	0.061	0.005 *	0.057	0.009 *

* *p* < 0.05: statistical significance. Notes: eLOS, extended length of hospital stay; BMI, body mass index; mECM, modified Elixhauser’s Comorbidity Measure.

## Data Availability

All the data supporting the study findings are within the manuscript. Additional detailed information and raw data are available from the corresponding author (Junfei Guo) on reasonable request.

## Abbreviations

IF	Intertrochanteric fractures
eLOS	Extended length of hospital stay
IMN	Intramedullary fixation
PFNA	Proximal femoral nail antirotation
BMI	Body mass index
mECM	Modified Elixhauser comorbidity method
NRS	Numerical rating scores
GDS	Geriatric Depression Scale
FIM	Functional independence measure
OR	Odds ratio
CI	Confidence interval
